# Pollen DNA metabarcoding identifies regional provenance and high plant diversity in Australian honey

**DOI:** 10.1002/ece3.7679

**Published:** 2021-06-03

**Authors:** Liz Milla, Kale Sniderman, Rose Lines, Mahsa Mousavi‐Derazmahalleh, Francisco Encinas‐Viso

**Affiliations:** ^1^ Centre for Australian National Biodiversity Research CSIRO Canberra ACT Australia; ^2^ School of Earth Sciences The University of Melbourne Melbourne Vic. Australia; ^3^ eDNA Frontiers Laboratory Curtin University Perth WA Australia

**Keywords:** bee nutrition, biomonitoring, European honeybee, ITS2, metabarcoding, molecular diet analysis, plant DNA, trnL

## Abstract

Accurate identification of the botanical components of honey can be used to establish its geographical provenance, while also providing insights into honeybee (*Apis mellifera* L.) diet and foraging preferences. DNA metabarcoding has been demonstrated as a robust method to identify plant species from pollen and pollen‐based products, including honey. We investigated the use of pollen metabarcoding to identify the floral sources and local foraging preferences of honeybees using 15 honey samples from six bioregions from eastern and western Australia. We used two plant metabarcoding markers, ITS2 and the trnL P6 loop. Both markers combined identified a total of 55 plant families, 67 genera, and 43 species. The trnL P6 loop marker provided significantly higher detection of taxa, detecting an average of 15.6 taxa per sample, compared to 4.6 with ITS2. Most honeys were dominated by *Eucalyptus* and other Myrtaceae species, with a few honeys dominated by *Macadamia* (Proteaceae) and Fabaceae. Metabarcoding detected the nominal primary source provided by beekeepers among the top five most abundant taxa for 85% of samples. We found that eastern and western honeys could be clearly differentiated by their floral composition, and clustered into bioregions with the trnL marker. Comparison with previous results obtained from melissopalynology shows that metabarcoding can detect similar numbers of plant families and genera, but provides significantly higher resolution at species level. Our results show that pollen DNA metabarcoding is a powerful and robust method for detecting honey provenance and examining the diet of honeybees. This is particularly relevant for hives foraging on the unique and diverse flora of the Australian continent, with the potential to be used as a novel monitoring tool for honeybee floral resources.

## Graphical abstract

We used pollen DNA metabarcoding to identify the botanical content of 15 honey samples from six Australian biogeographical regions. We sequenced ITS2 and trnL P6 loop, which in combination identified 55 plant families, 67 genera, and 43 species. The trnL marker was able to differentiate between eastern and western Australian honeys and cluster samples into biogeographical regions.

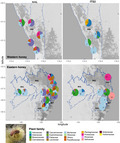

## 1 INTRODUCTION

European honeybees (*Apis mellifera* L.) are one of the most important pollinators of crops worldwide (Calderone, 2012; Potts et al., 2010), as well as the principal producers of commercial honey and pollen products (Pascoal et al., 2014). Recently, interest has grown in improved hive management practices due to large reported declines in global colony numbers (Dainat et al., 2012; Ellis et al., 2010; Seitz et al., 2015; vanEngelsdorp et al., 2010). Many hive failures are attributed to colony collapse disorder (Johnson et al., 2010; Ratnieks & Carreck, 2010; vanEngelsdorp et al., 2009), a phenomenon driven largely by the interaction between the mite *Varroa destructor* and deformed wing virus (Dahle, 2015; Dainat et al., 2012; Guzmán‐Novoa et al., 2010), and exacerbated by other stressors such as poor nutrition (Roberts et al., 2017). Accordingly, identifying and managing the nutritional needs of hives by providing adequate floral resources is one of the recommendations to improve honeybee health and help reduce colony loss (Goulson et al., 2015; Mao et al., 2013).

The nutritional intake of honeybees comes from plant nectar and pollen (Brodschneider & Crailsheim, 2010). Studies of honeybee diets have suggested a diversity of pollen can lead to benefits such as increased lifespan and improved immunocompetency (Alaux et al., 2010; Pasquale et al., 2013). Therefore, a lack of suitably diverse foraging habitat, potentially in decline due to factors such as climate change, intensive farming practices, and urbanization, could lead to poor nutritional outcomes for bees (Donkersley et al., 2014; Goulson et al., 2015; Naug, 2009). While the foraging distance of honeybees can reach several kilometers, it may be as short as a few hundred meters depending on the resources available, the season and surrounding landscape structure (Beekman & Ratnieks, 2000; Couvillon et al., 2015; Danner et al., 2017). Consequently, the availability of suitable foraging plants is likely to vary considerably between individual hive locations. As anecdotal evidence of bee foraging can be unreliable (Garbuzov & Ratnieks, 2014), empirical methods to identify foraged plants are preferable. Honey is a direct product of honeybee foraging; thus, it is highly suitable to study their long‐term nutritional needs. Pollen derived from honey can easily capture longer foraging intervals than pollen traps, commonly used plastic enclosures that remove pollen pellets from the corbiculae as bees enter the hive, impacting hive health and foraging activity when used for extended periods (Dubois et al., 2018; Webster et al., 1985). Microscopy‐based pollen identification methods have been widely applied to establish the geographical provenance and floristic origin of honey (Von Aronne & Micco, 2010; der Ohe et al., 2004) and as a quality control protocol, particularly in the European Union (EU Directive Council, 2001). The traditional microscopy method, known as melissopalynology, is a time‐consuming task requiring considerable expertise and knowledge of local flora and often cannot provide taxonomic resolution beyond family or genus level (Khansari et al., 2012; Sniderman et al., 2018; Thornhill & Crisp, 2012). Melissopalynology has only been recently applied to examine the pollen composition of Australian honey in order to determine its geographical provenance. In their study, Sniderman et al. (2018) found that the current palynological criteria used to verify the origin of *Eucalyptus* honeys in Europe could not be relied upon for Australian products, as the palynological profile of Australian *Eucalyptus* honey was substantially different to that of southern European *Eucalyptus* honey. They determined that this discrepancy was mainly due to the presence of species indigenous to Australia. Consequently, the nutritional needs of bee colonies in countries with a unique and diverse flora such as Australia are likely to be met by a very different suite of plants than those found in Europe or North America.

Pollen DNA metabarcoding, a molecular method for pollen identification, has been used several times to investigate foraging by bees (Cornman et al., 2015; Galimberti et al., 2014; Nürnberger et al., 2019; Richardson, Lin, Quijia, et al., 2015; de Vere et al., 2017; Wilson et al., 2010) and to establish the floral content and geographical provenance of honey (Bruni et al., 2015; Hawkins et al., 2015; Utzeri et al., 2018). Pollen DNA metabarcoding is an easily scalable method that can detect higher numbers of taxa than microscopy (Keller et al., 2015; Smart et al., 2017), with greater resolution and repeatability of results (Hawkins et al., 2015). However, there are no individual genetic markers that can discriminate all known plant diversity (Hollingsworth et al., 2009, 2011), and estimating the proportions of taxa present in samples with DNA metabarcoding can be challenging (Baksay et al., 2020; Piñol et al., 2019), for example, due to DNA extraction or PCR biases (Bell et al., 2016; Pornon et al., 2016). Nonetheless, the advantages of molecular over traditional methods can be significant. For example, as honey contains DNA from both pollen‐ and nectar‐providing plants (Prosser & Hebert, 2017), molecular methods can identify a broader range of plants in a colony's diet than pollen analysis alone. Previous metabarcoding studies from honey have revealed that while honeybees are generalists, only a small proportion of the flowering taxa seems to be abundant in their diet, with many species present in small proportions (Hawkins et al., 2015; de Vere et al., 2017). While care must be taken to remove false positives and background contamination that are commonly found in metabarcoding results (Ficetola et al., 2015), plant taxa present in very low proportions could represent an important nutritional contribution for overall health (Pasquale et al., 2013; Requier et al., 2015). Metabarcoding studies have also demonstrated that pollen foraged by honeybees can reflect the local plant biodiversity and flowering phenology (Cornman et al., 2015; Galimberti et al., 2014), and could be used to detect and monitor the presence of plant species of interest (Bell, de Vere, et al., 2016; Richardson, Lin, Sponsler, et al., 2015; Tremblay et al., 2019).

Most pollen metabarcoding studies, including those using honey pollen, have been conducted in European or North American countries. There are very few pollen metabarcoding studies from Australia (Elliott et al., 2021; McFrederick & Rehan, 2019), where there are many endemic plant species. Australian landscapes are currently divided into 89 large geographically distinct bioregions, based on common factors such as climate, geology, landform, and native vegetation, through a system known as the Interim Biogeographical Regionalisation for Australia (IBRA) (Thackway & Cresswell, 1997). In this study, we use pollen metabarcoding to examine the floral content of 15 commercially produced honeys from eastern and western Australia, sourced from twelve localities within six different IBRA regions, and previously characterized via melissopalynology by Sniderman et al. (2018). We chose two commonly used plant metabarcoding markers, ribosomal internal transcribed region 2 (ITS2), and the P6 loop region of the transfer RNA gene for Leucine (trnL). ITS2 has been shown to provide good discriminatory power in pollen metabarcoding studies, particularly at the genus level (Keller et al., 2015; Richardson et al., 2019; Richardson, Lin, Sponsler, et al., 2015). The trnL P6 loop is a very short chloroplast region with high amplification success, and it has been effective in detecting plant taxa in honey (Pornon et al., 2016; Valentini et al., 2010). We first compare the plant taxonomic resolution provided by each of the two metabarcoding markers and then compare the combined identifications to a previous study of the same honey samples using light microscopy. Finally, we also examine whether pollen found in honey reflects the flora from its IBRA region of origin, and determine the usefulness of pollen metabarcoding in discriminating between bioregions, and in detecting plant species from a particular locality.

## 2 MATERIALS AND METHODS

### 2.1 Honey selection

We selected 15 honey samples from twelve localities located within six Australian IBRA regions. The raw honey samples were collected in January (*n* = 3), September (*n* = 1), and October (*n* = 11) 2015 (Table 1). Each sample was collected in clean food‐grade containers filled directly from beekeeper extractions from single apiaries, and sampling equipment was cleaned between each collection to avoid cross‐contamination as detailed in Sniderman et al. (2018). Samples were stored at room temperature until 2019, when they were transferred to a −20°C freezer. To take a subsample, the containers were first placed in a water bath at 40°C for 30 min to reduce viscosity. Between 6.4 and 11 g of honey were taken from each container using a sterile large syringe, and transferred to sterile 50‐ml tubes, which were stored at −20°C until DNA extraction.

**TABLE 1:** Australian honey samples analyzed in this study

Australian region	Sample ID	Approximate locality	State	IBRA region	Producer nominated source	Collected Mon‐YY	Sample weight (g)	Total DNA (ng)
West	SJ1	Yanchep	WA	SWA	Spring Eucalypt	Oct‐15	4.024	12.375
West	SJ2	Wanneroo	WA	SWA	Mild spring Eucalypt	Oct‐15	3.947	10.88
West	SJ3	Gingin	WA	SWA	Spring Eucalypt	Jan‐15	4.936	10.215
West	SJ4	Lancelin	WA	SWA	Spring Eucalypt	Jan‐15	4.506	11.595
West	SJ5	Lancelin	WA	SWA	Spring Eucalypt	Jan‐15	4.311	11.585
East	SJ6	Cunnamulla	Qld	MUL	Yapunyah	Oct‐15	4.355	11.485
East	SJ7	Tingha	NSW	NET	Canola	Oct‐15	4.476	11.635
East	SJ8	Cunnamulla	Qld	MUL	Yapunyah	Oct‐15	3.808	11.885
East	SJ9	Warwick	Qld	BBS	Mixed Honey	Oct‐15	4.176	6.955
East	SJ10	Warwick	Qld	BBS	Macadamia	Oct‐15	3.3	7.84
East	SJ11	Tamworth	NSW	NAN	White Box/Mixed honey	Sep‐15	4.513	7.3
East	SJ12	Blackstone	Qld	SEQ	Blue Gum/Mixed Honey	Oct‐‐15	3.837	7.325
East	SJ13	Bundaberg	Qld	SEQ	Macadamia	Oct‐15	3.52	8.175
East	SJ14	Maclean	Qld	SEQ	Mixed Honey/Macadamia	Oct‐15	3.887	7.87
East	SJ15	Lismore	NSW	SEQ	Macadamia	Oct‐15	3.644	7.565

Note

Interim Biogeographic Regionalisation for Australia (IBRA) codes listed are as follows: BBS = Brigalow Belt South, MUL = Mulga Lands, NAN = Nandewar, NET = New England Tablelands, SEQ = South East Queensland, SWA = Swan Coastal Plain. Collected Mon‐YY refers to month and year honey was sourced by producer, Sample weight (g) refers to subsample weight used for DNA extraction, and Total DNA (ng) refers to weight of DNA from subsample extraction.

### 2.2 DNA extraction and sequencing

DNA extractions from each subsample were conducted in November 2019 and March 2020. The pollen isolation protocol for honey was modified from de Vere et al. (2017). Samples were diluted with sterile water to make up to 15 ml. These mixtures were incubated for 30 min in a water bath at 65°C, with occasional vortexing. After incubation, mixtures were centrifuged for 15 min at 7,000 rpm at room temperature. The supernatant was discarded and the remaining pellet resuspended in 400 μl of lysis buffer (CF buffer in the Macherey‐Nagel DNA Food extraction kit) and transferred to a 1.5‐ml tube, to which 20 μl of Proteinase K was added. The samples, including a blank lysis control (consisting of 400 μl buffer CF and 20 μl Proteinase K), were then incubated in a shaker oven at 65°C for 1 hr. DNA extraction then proceeded as per the manufacturer's protocol (Macherey‐Nagel). Final DNA concentrations were quantified using a Quantus (Promega Corporation) fluorometer.

### 2.3 DNA metabarcoding and sequencing

An initial PCR was performed in duplicate reactions on each DNA extraction, to determine the DNA dilution for optimal amplification by adding DNA template either directly to the PCR master mix, or as a 1/10 dilution. PCR master mixes comprised: 2.5 mM MgCl_2_ (Applied Biosystems), 10× PCR Gold buffer (Applied Biosystems), 0.25 mM dNTPs (Astral Scientific, Australia), 0.4 mg/ml bovine serum albumin (Fisher Biotec), 0.4 μmol/l forward and reverse primers, 0.6 μl of a 1:10,000 solution of SYBR Green dye (Life Technologies), and 1 U AmpliTaq Gold DNA polymerase (Applied Biosystems). All PCRs had a volume of 25 µl and were performed using StepOne Plus Instruments (Applied Biosystems). PCR cycling conditions consisted of denaturation at 95°C for 5 min, followed by 50 cycles of: 95°C for 30 s, 52°C (trnL) or 55°C (ITS2) for 30 s, 72°C for 45 s, finishing with a final extension stage at 72°C for 10 min.

Indexing of samples was achieved using unique, single use combinations of 8 bp multiplex identifier‐tagged (MID‐tag) primers as described in Koziol et al. (2019) and van der Heyde et al. (2020). MID‐tag PCRs were prepared using a Qiagility instrument (Qiagen) using the same master mix and PCR conditions as described. Negative and positive PCR controls for both trnL and ITS2 were included to ensure validity of results. Sequencing libraries were pooled equimolarly based on the PCR amplification results. Libraries were size selected using a Pippin instrument (Sage Sciences), quantified on a Qubit (Thermo Fisher), and diluted to 2nM. Libraries were sequenced on an Illumina MiSeq instrument using 300 cycle V2 kit (trnL) or 500 cycle kit (ITS2) with custom sequencing primers.

### 2.4 Reference database construction

Sequence reference databases containing taxa known to occur in the locality can improve the taxonomic resolution of metabarcoding results by reducing the number of potentially erroneous matches (Tab erlet et al., 2006). For this study, the localities given as the honey source were given only by name, to which approximate coordinates were assigned by Sniderman et al. (2018). To construct local reference databases for ITS2 and trnL, we first downloaded all plant records from the six IBRA regions where the localities were found from the Atlas of Living Australia (www.ala.org, Table 2) and filtered each list to species that had at least three records from the area since 1980. We used a custom R (R Core Team, 2019) script to search for species by name in ITS2DB (Ankenbrand et al., 2015) and PlantAligDB trnL_GH (Santos et al., 2019), both downloaded 26 August 2020, and to generate FASTA files with taxonomy lineage headers. Searchable databases were created from the resulting sequence reference files using the USEARCH (Edgar, 2010) makeudb program.

**TABLE 2:** DOI numbers for Atlas of Living Australia (ALA) record downloads used to construct taxonomy database (www.ala.org)

IBRA Region	Code	DOI
Brigalow Belt South	BBS	10.26197/ala.c42165a0‐fcc6‐4552‐a28a‐74d0978e582b
Mulga Lands	MUL	10.26197/ala.67738621‐dcb0‐4346‐a6f6‐53f2cbbe82e3
Nandewar	NAN	10.26197/ala.e708fa9d‐54bd‐4936‐‐95cf‐767d967e6e57
New England Tablelands	NET	10.26197/ala.bac1a9c1‐3133‐4e49‐b37d‐ca0d8fca2a12
South Eastern Queensland	SEQ	10.26197/ala.3198417c‐2a27‐43d0‐9c43‐c9c2fd81d036
Swan Coastal Plain	SWA	10.26197/ala.77257667‐f711‐4aac‐b345‐6cdf24a4d7c6

### 2.5 Data processing and taxonomy assignment

Adapters were removed from raw sequencing reads using AdapterRemoval (Schubert et al., 2016), and trimmed reads filtered to quality ≥20 using the USEARCH fastq_filter function. Additionally, for paired‐end ITS2, reads 1 and 2 were merged by setting the minimum overlapping sequence length to 13. OBITools (Boyer et al., 2016) was used to demultiplex all samples. We then used OBITools and mothur (Schloss et al., 2009) to remove ITS2 sequences smaller than 200 and >450 bp, and trnL sequences <30 and >180 bp. Clean sequencing reads were grouped into molecular taxonomic units (MOTUs), representing putative individual species. We merged unique reads with the USEARCH fastqx_uniques function and generated denoised MOTUs (zero‐noise operational taxonomic units, or ZOTUs) with the unoise3 algorithm. We mapped the filtered reads back to add read counts per ZOTU. To identify each ZOTU, we searched the ZOTUs against the custom databases using the USEARCH sintax command with a 90% bootstrap cutoff. We also searched all sequences against the NCBI nt database with an e‐value of 1e−5. The top five BLAST results were parsed using the python script taxonomy_assignment_BLAST_V1.py (github: Joseph7e/Assign‐Taxonomy‐with‐BLAST), which assigns the lowest level of taxonomic identification (species < genus < family) from the results. We compared the results against the USEARCH results and removed ZOTUs where the family or genus assigned by USEARCH differed from the consensus BLAST taxonomy.

### 2.6 Analysis of honey composition and comparison to microscopy results

We used a custom R script to run statistical analyses. For ZOTUs without a family assignment from the custom database, we assigned the BLAST match of ≥99% probability if considered to be a realistic match. We checked all samples for potential contamination by checking against the negative controls, and we checked for adequate sampling depth using rarefaction analysis with the rarecurve function of the vegan package (Oksanen et al., 2007).

We merged all ZOTUs to their lowest common taxonomic identification using the tax_glom function of the phyloseq R package (McMurdie & Holmes, 2013). This is likely to underestimate the real numbers of taxa in the metabarcoding results, as individual ZOTUs can represent different species that could not be differentiated, and thus, pooling them to a common ancestor does not reflect the true diversity. However, ZOTUs can also represent the same species within the same marker or across markers; for example, several different ITS2 ZOTUs were identified by BLAST as *Glycine max*. Therefore, we chose a conservative approach for the number of taxa found by metabarcoding. Additionally, we filtered out low‐abundance taxa represented by <0.01% of the reads within each sample. We then counted the total numbers of taxa detected in each sample and used a Wilcoxon rank‐sum test to determine whether there were differences in species richness between eastern and western regions, and between metabarcoding markers. To calculate relative read abundances, which is the proportion of reads for each taxon present in a sample out of the total reads for that sample, we used the decostand function of the vegan R package (Oksanen et al., 2007). We chose the best dissimilarity index by calculating the rank‐order similarity with gradient separation using the rankindex function of vegan. We then calculated distance matrices of relative abundances using the vegdist function of vegan using the highest ranked dissimilarity index from rankindex for each marker. We also examined these differences via PERMANOVA analysis with 999 permutations using the adonis function from vegan. We repeated the PERMANOVA analysis using presence/absence data (i.e., if relative abundance in sample >0, then taxon was recorded as present, else absent). We visualized the differences for the eastern and western regions using a Constrained Analysis of Principal Coordinates (CAP) and Horn's distance with the ordinate function of the phyloseq R package. We also performed hierarchical unweighted pair group method with arithmetic mean (UPGMA) clustering analysis using the hclust function in R.

To compare the numbers of taxa from metabarcoding to the microscopy data from Sniderman et al. (2018), we combined the identifications from trnL and ITS2 to every level of taxonomic identification (family, genus, species, and taxon). To compare identifications at taxon level, we considered each pollen morphospecies identified in the microscopy results as equivalent to a taxon, see Table S1. As with the metabarcoding data, we filtered out taxa represented by <0.01% of the pollen grains within each sample. We used a one‐sided Wilcoxon rank‐sum test to determine whether the numbers of taxa detected by metabarcoding were higher than microscopy at every level of taxonomic identification. We excluded species‐level results from further analyses as only one species (*Trifolium repens*) was identified via microscopy. We compared the overlap in families and genera detected with both methods for each sample, and for all samples combined. We also examined the composition of honey samples by comparing the top five taxa and top five families by abundance, using either total pollen grain proportion for microscopy or relative read abundance for metabarcoding markers. From the top five taxa and families, we counted the number of matches found via metabarcoding to the microscopy results to determine the overlap in detection of the most abundant taxa. We then compared the top five taxa and families to the nominated floral source of each honey sample, to determine how each method performed in detecting the main expected pollen type.

## 3 RESULTS

### 3.1 Taxonomic assignment and marker comparison

The list of plant species recorded within all six IBRA regions with more than three records since 1980 contained a total of 8,679 species. Of these, 2,551 were from western localities and 6,729 were from eastern localities, with 601 species recorded in both regions. Of all the recorded species, we found a reference sequence for 2,342 species (26.9%) in the ITS2DB database, and for 1,320 species (15.2%) in the trnL_GH database. The total number of sequences added to the local custom database was 14,017 for ITS2 and 1,864 for trnL, which included several representatives for some species, including subspecies and varieties.

From the sequencing data, we obtained 1,731,431 filtered reads for ITS2, and 1,365,187 for trnL. The number of reads per honey sample ranged from 154 to 181,787 for ITS2, and from 41,529 to 118,774 for trnL. In total, 93.9% of filtered ITS2 reads were matched to the custom database to at least family level, and a further 5.2% were assigned a match using BLAST. Most of the ITS2 ZOTUs identified only by BLAST were matched to *Populus deltoides* (Eastern cottonwood) and *G. max* (soybean). For trnL, 90.9% filtered reads were matched to the custom database to at least family level, and a further 4.7% were assigned a match using BLAST. The trnL ZOTUs identified only by BLAST were matched to a diverse range of plants, with the most abundant ZOTUs identified as *Casuarina*, *Crocus*, Solanaceae, and *Viburnum*. Based on the rarefaction analysis, we removed sample SJ12 (154 total reads) from the ITS2 results. After merging to lowest assigned taxonomic identifications, we merged 386 ITS2 ZOTUs to 27 unique taxa and 140 trnL ZOTUs to 83 unique taxa.

**FIGURE 1:**
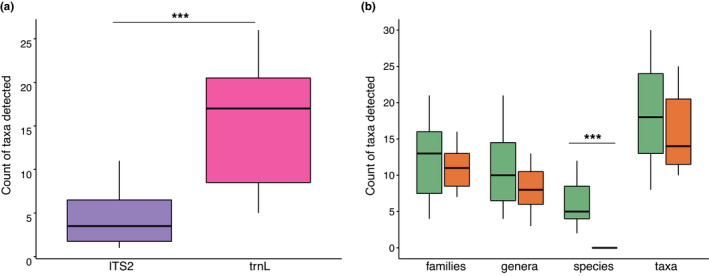
(a) Comparison of taxa detected per sample by metabarcoding markers different levels of identification. Purple colour represents ITS2, pink represents trnL. (b) Comparison of taxa detected per sample by combined metabarcoding markers and microscopy at different levels of identification. Green colour represents metabarcoding results and orange represents microscopy results

We identified significantly more taxa per sample with trnL than with ITS2 (mean trnL = 15.6, ITS2 = 4.6; Wilcoxon rank‐sum test, two‐tailed, *p* < 0.001; Figure 1a). While the total numbers of identified taxa differed greatly, we found a high overlap between the number of families detected by both ITS2 and trnL (eight of 10 families detected by ITS2 also detected by trnL). However, the overlap between the genera identified by both markers was low (4 of 16 genera found by ITS2 also found by trnL). This result is partly due to the differences in taxonomic resolution by each marker, but may also represent marker amplification bias. A total of 103 taxa (Table S2) were identified by both markers combined, with 43 identified to species level, 33 to genus level and 27 to family level. Overall, we detected more distinct species (trnL: 31, ITS2: 13), genera (trnL: 55, ITS2: 16), and families (trnL: 53, ITS2: 10) with trnL than ITS2. Some important taxa, however, such as *Eucalyptus,* could only be detected by ITS2 at the genus or species level.

### 3.2 Pollen composition and provenance analysis

**FIGURE 2:**
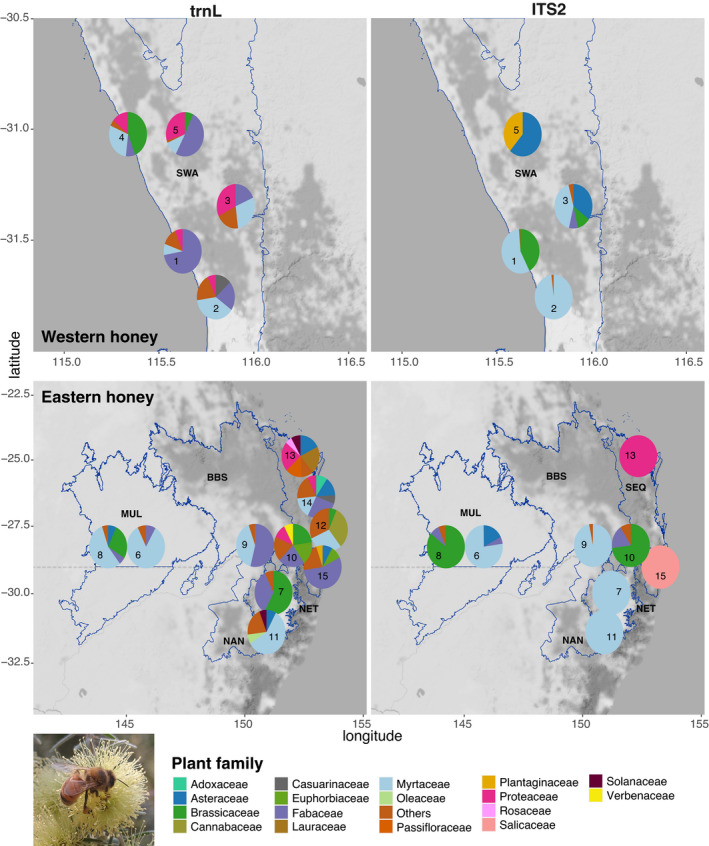
Relative abundance of plant families found in 15 honey samples by trnL (left panels) and ITS2 (right panels). Families of taxa found at abundances <5% are grouped into “Others.” Sample names are shown without “SJ” prefix. Blue lines indicate IBRA bioregion boundaries: BBS = Brigalow Belt South, MUL = Mulga Lands, NAN = Nandewar, NET = New England Tablelands, SEQ = South East Queensland, SWA = Swan Coastal Plain

The mean number of taxa detected by the combination of trnL and ITS2 per honey sample was 18.1 (range from 8 to 30). The most commonly detected taxa in honey samples were (number of samples) undifferentiated species of Fabaceae (15), Asteraceae (13), Myrtaceae (12), and Brassicaceae (10), followed by *Macadamia integrifolia* (9), and *Melaleuca nodosa* (9). The top plant families by relative abundance found in honey samples by each marker are shown in Figure 2.

The predominant floral source given by the beekeeper/producer was detected at genus or family level in the top five most abundant taxa for 12 of the 14 honeys (Table 3). For nine of the samples, generic eucalypt (alternatively labeled as White Box, Spring Eucalypt, Yapunyah, and Blue Gum) was nominated as the primary floral source. In the ITS2 metabarcoding results, we considered both *Eucalyptus* and *Corymbia* as positive identifications for samples labeled as eucalypt. For trnL, the lack of resolution for any *Eucalyptus* species meant that results were likely contained within Myrtaceae sp., and we considered this a positive identification. *Macadamia integrifolia* was listed as the second most common predominant floral source of pollen and nectar, nominated for four honeys from Queensland and New South Wales (SJ10, SJ13, SJ14, and SJ15). It was detected in the highest abundance in those four samples by the trnL results. *Macadamia* was also detected in another eastern sample (SJ12, trnL: 1.28%) as well as in four western samples, albeit in very low proportions (trnL: <0.08%).

**TABLE 3:** Top five taxa by relative abundance detected by metabarcoding and microscopy (relative proportion of identified reads—metabarcoding, or relative proportion of grains—microscopy, in brackets) in each honey sample

Region	ID	Description	trnL	ITS2	Microscopy
West	SJ1	Spring Eucalypt, WA	Fabaceae sp. (0.723), **Myrtaceae sp. (0.081),** Proteaceae sp. (0.065), *Pelargonium* sp. (0.033), *Grevillea* sp. (0.031)	***Corymbia*** **sp. (0.566),** Brassicaceae sp. (0.421), *Brassica* sp. (0.013)	***Corymbia sp. (0.376)*, *Eucalyptus*** **sp. (0.256),** *Acacia* sp. (0.106), *Leucopogon* sp. (0.073), *Banksia* sp. (0.064)
West	SJ2	Mild spring Eucalypt, WA	*Melaleuca nodosa* (0.259), Fabaceae sp. (0.214), *Casuarina* sp. (0.133), **Myrtaceae sp. (0** . **12),** Proteaceae sp. (0.073)	Myrtaceae sp. (0.981), *Jacksonia horrida* (0.015), Brassicaceae sp. (0.005)	***Eucalyptus*sp. (0.518), *Corymbia*** **sp. (0.128)**, Fabaceae sp. (0.063), *Leucopogon* sp. (0.061), *Banksia* sp. (0.056)
West	SJ3	Spring Eucalypt, WA	Proteaceae sp. (0.323), **Myrtaceae sp. (0** . **242),** Fabaceae sp. (0.186), *Melaleuca* *nodosa* (0.052), *Daviesia ulicifolia* (0.031)	Myrtaceae sp. (0.418), Asteraceae sp. (0.246), Brassicaceae sp. (0.113), *Arctotheca* sp. (0.105), *Daviesia divaricata* (0.075)	***Eucalyptus*** **sp. (0.413),** *Echium* sp. (0.2), ***Corymbia*** **sp. (0.107),** *Banksia* sp. (0.076), Leptospermeae sp. (0.066)
West	SJ4	Spring Eucalypt, WA	Brassicaceae sp. (0.338), **Myrtaceae sp. (0.295)**, Proteaceae sp. (0.142), *Diplotaxis tenuifolia* (0.101), Fabaceae sp. (0.079)		***Eucalyptus*sp. (0.473),*Corymbia*** **sp. (0.295),** Brassicaceae sp. (0.082), Oleaceae sp. (0.078), *Banksia* sp. (0.021)
West	SJ5	Spring Eucalypt, WA	Fabaceae sp. (0.508), *Grevillea* sp. (0.186), Proteaceae sp. (0.11), **Myrtaceae sp. (0.107),** Brassicaceae sp. (0.071)	Asteraceae sp. (0.616), *Plantago lanceolata* (0.384)	***Eucalyptus*sp. (0.533), *Corymbia*** **sp. (0.252),** Brassicaceae sp. (0.064), *Acacia* sp. (0.044), *Banksia* sp. (0.04)
East	SJ6	Yapunyah	**Myrtaceae sp. (0.844),** Fabaceae sp. (0.083), *Casuarina* sp. (0.038), Asteraceae sp. (0.022), Scrophulariaceae sp. (0.013)	***Eucalyptus*sp. (0.77)**, Asteraceae sp. (0.175), *Glycine* *max* (0.052), Fabaceae sp. (0.001)	***Eucalyptus*** **sp. (0.937),** Tubuliflorae sp. (0.021), Brassicaceae sp. (0.013), Myoporeae sp. Large (0.013), Myoporeae sp. Small (0.005)
East	SJ7	Canola	**Brassicaceae sp. (0.526),** *Vicia* sp. (0.343), *Diplotaxis tenuifolia* (0.055), Fabaceae sp. (0.029), Myrtaceae sp. (0.027)	*Eucalyptus* sp. (1)	**Brassicaceae sp. (0.695),** *Eucalyptus* sp. (0.17), *Vicia* sp. (0.106), *Centaurea* sp. Centaurea (0.008), *Corymbia* sp. (0.005)
East	SJ8	Yapunyah	**Myrtaceae sp. (0.554),** Brassicaceae sp. (0.269), Asteraceae sp. (0.07), Fabaceae sp. (0.055), Scrophulariaceae sp. (0.042)	*Brassica* sp. (0.503), Brassicaceae sp. (0.352), *Glycine* *max* (0.079), ***Eucalyptus*** **sp. (0.046),** *Trifolium* *repens* (0.012)	***Eucalyptus*sp. (0.483), *Corymbia*** **sp. (0.227),** Myoporeae sp. Large (0.196), Brassicaceae sp. (0.035), Tubuliflorae sp. (0.03)
East	SJ9	Mixed Honey	*Trifolium* sp. (0.416), Myrtaceae sp. (0.405), Fabaceae sp. (0.124), Brassicaceae sp. (0.024), Solanaceae sp. (0.017)	*Eucalyptus* sp. (0.819), Myrtaceae sp. (0.149), Fabaceae sp. (0.021), *Pisum* sp. (0.01)	*Eucalyptus* sp. (0.58), *Acacia* sp. (0.271), Brassicaceae sp. (0.071), Goodeniaceae sp. (0.039), *Polygonum* sp. (0.019)
East	SJ10	Macadamia	Brassicaceae sp. (0.227), *Mallotus* sp. (0.176), *Trifolium* sp. (0.148), ***Macadamia integrifolia*** **(0.107),** Verbenaceae sp. (0.078)	*Brassica* sp. (0.635), *Trifolium* *repens* (0.096), Brassicaceae sp. (0.095), Fabaceae sp. (0.082), *Trifolium* sp. (0.047)	Brassicaceae sp. (0.414), ***Macadamia*** **sp. (0.275),** *Eucalyptus* sp. (0.103), Tubuliflorae sp. (0.052), *Vicia* sp. (0.043)
East	SJ11	White Box/Mixed honey	**Myrtaceae sp. (0.581),** Asteraceae sp. (0.082), Oleaceae sp. (0.069), Solanaceae sp. (0.062), *Medicago sativa* (0.04)	***Eucalyptus*sp. (1)**	***Eucalyptus*** **sp. (0.917),** ***Corymbia*** **sp. (0.03)**, *Centaurea* sp. Centaurea (0.018), Brassicaceae sp. (0.014), Tubuliflorae sp. (0.007)
East	SJ12	Blue Gum/Mixed Honey	Cannabaceae sp. (0.326), **Myrtaceae sp. (0.177)**, *Melaleuca* *nodosa* (0.114), Brassicaceae sp. (0.066), Verbenaceae sp. (0.05)		***Eucalyptus*** **sp. (0.332),** *Macadamia* sp. (0.258), Rutaceae sp. (0.088), *Acacia* sp. (0.072), Undetermined sp. Monocot (0.048)
East	SJ13	Macadamia	***Macadamia integrifolia*** **(0.232),** Lauraceae sp. (0.211), Asteraceae sp. (0.176), *Passiflora* sp. (0.141), *Capsicum* sp. (0.082)	***Macadamia*sp. (1)**	***Macadamia*** **sp. (0.526),** *Eucalyptus* sp. (0.195), Fabaceae sp. (0.081), *Trifolium* sp. (0.054), *Corymbia* sp. (0.025)
East	SJ14	Mixed Honey/Macadamia	*Melaleuca* *nodosa* (0.162), Asteraceae sp. (0.145), *Trifolium* sp. (0.142), Fabaceae sp. (0.124), *Viburnum* sp. (0.096), ^a^ ***Macadamia integrifolia*(0.075)**		*Eucalyptus* sp. (0.499), Fabaceae sp. (0.119), ***Macadamia*** **sp. (0.106),** Elaeocarpaceae sp. (0.075), Tubuliflorae sp. (0.05)
East	SJ15	Macadamia	*Trifolium* sp. (0.391), *Austrocallerya australis* (0.107), *Mallotus* sp. (0.094), Asteraceae sp. (0.083), *Plantago lanceolata* (0.055), ^a^ ***Macadamia integrifolia*(0.021)**	*Populus deltoides* (1)	***Macadamia*** **sp. (0.356),** *Eucalyptus* sp. (0.17), Fabaceae sp. (0.142), *Trifolium* sp. (0.084), Tubuliflorae sp. (0.076)

Note

Bold underlined type indicates where the main floral source nominated by the producer has been detected. For Eucalypt/White Box/Yapunyah/Blue Gum, we highlight Myrtaceae sp. for trnL, and *Corymbia* sp. and *Eucalyptus* sp. for ITS2.

^a^ Taxon detected outside of the top five.

One of the honeys (SJ7 from Tingha, NSW) was nominally derived from Canola (*Brassica napus*, Brassicaceae), and this was also the sample with the highest proportion of Brassicaceae reads for trnL (52.6%). *Brassica* species were more common in the eastern honeys than in the west. Proteaceae are a family predominantly distributed in the Southern Hemisphere, with rich diversity found in south‐western Australia (Collins & Rebelo, 1987). Higher proportions of Proteaceae (with the exception of *Macadamia integrifolia*) were detected in western honeys. For example, *Grevillea* was common in the western samples, particularly in one of the honeys from Lancelin (SJ5, trnL: 18.62%), but rarely found in the eastern honeys (one only, SJ10, trnL: 0.15%). An eastern Myrtaceae species, *Melaleuca nodosa*, was found in substantial proportions (>2.5%) in six honeys, including two western Australian honeys (SJ2 trnL: 25.92% and SJ3 trnL: 5.19%). There was only one *Melaleuca* reference sequence in the trnL custom database (and no relevant hit from the NCBI nt database); therefore, it is likely that this species is instead a western species of *Melaleuca*.

**FIGURE 3:**
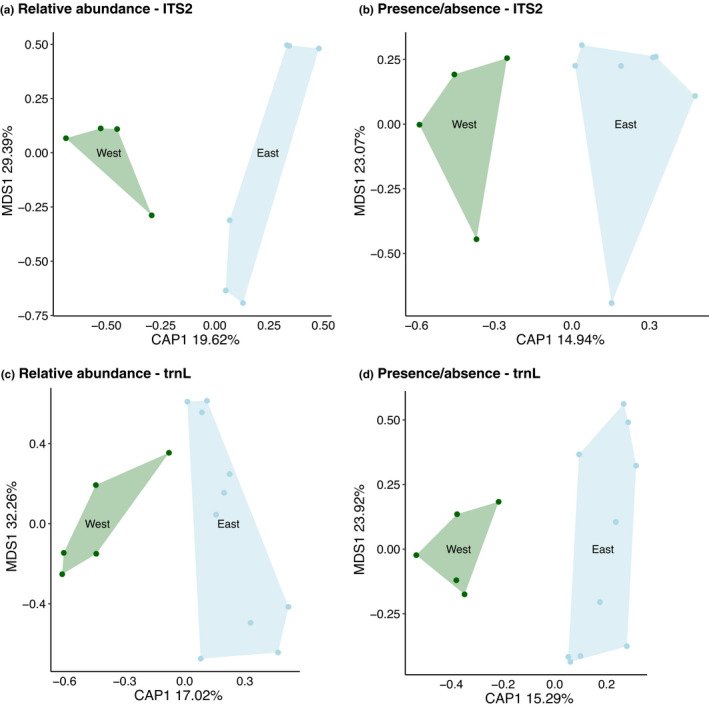
PCoA plots for ITS2 and trnL generated using Constrained Analysis of Principal Coordinates (CAP) and Horn's distance. Green represents Western Australia honey samples, and blue represents Eastern Australia honey samples. (a) ITS2 using relative read abundance per sample values, (b) ITS2 using presence/absence values, (c) trnL using relative read abundance per sample values, and (d) trnL using presence/absence values

There was no significant difference in the taxon richness of western and eastern honeys when combining the numbers of taxa detected by both markers (Wilcox rank‐sum test, *p* = 0.243). However, there was a close‐to‐significant difference in diet composition (beta diversity) based on the PERMANOVA dissimilarity analysis of the ITS2 results (index = Canberra, *p* = 0.059) and highly significant with trnL (index = Canberra, *p* = 0.001). The principal coordinate ordination analyses could clearly separate the eastern and western regions by relative abundance (Figure 3a,c) and presence/absence data (Figure 3b,d) using either marker.

**FIGURE 4:**
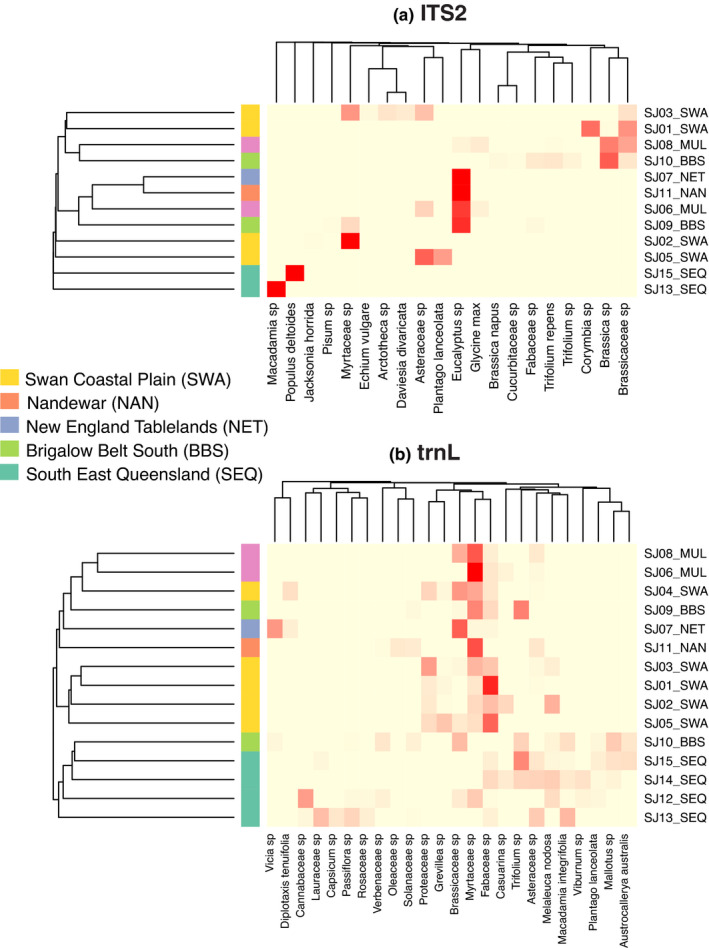
Heatmaps of beta diversity and clustering analysis. (a) ITS2. (b) trnL. Cells are colored by relative read abundance, and row dendrogram tips are color coded by IBRA bioregions. Plant taxa (*X*‐axis) are filtered to those present in abundances >1% for ITS2 and >5% for trnL

To determine whether the pollen composition of each honey reflected its regional origin, we compared the plants detected by metabarcoding to the list of plants from the corresponding IBRA region. We found the majority of genera detected in samples from a given region matched to genera found in the corresponding IBRA list (BBS: 74%, MUL: 75%, NAN: 100%, NET: 83%, SEQ: 86%, and SWA: 79%). At species level, the percentages of species found in the corresponding IBRA list were lower, particularly for Mulga Lands and New England Tablelands (BBS: 50%, MUL: 25%, NAN: 57%, NET: 33%, SEQ: 63%, and SWA: 40%). Some of the species detected in western honeys were commonly cultivated or invasive non‐natives, such as *B. napus* and *Plantago lanceolata*. However, some species detected in western honeys that occur mainly in eastern Australia, such as *Echium vulgare* (Boraginaceae), *Daviesia ulicifolia* (Fabaceae), and *M. nodosa* (Myrtaceae), were likely matched due to the lack of a western species reference for the same genus. In the eastern honeys, 23 plant species were identified from the eastern plant references, while five were identified by BLAST. In the western honeys, 10 species were found in the western references, while 14 were identified from the eastern list or BLAST. The clustering analysis for ITS2 based on relative abundances (Figure 4a) did not generate any clusters reflecting the region of origin. However, the corresponding clustering analysis for trnL (Figure 4b) showed a clear grouping for the Swan Coastal Plains (SWA, western region) samples with one exception (SJ4), and clustering of all samples from South East Queensland (SEQ, eastern region) with one sample from another IBRA region, Brigalow Belt South (SJ10). Mulga Lands (MUL, eastern regions) samples also clustered together, although this cluster is based on only two samples.

### 3.3 Comparison to microscopy results

The comparison of the number of taxa identified per sample at each taxonomic level shows that while the metabarcoding markers combined detect a higher mean number of taxa than microscopy at all taxonomic levels, these differences were not significant at family (Wilcoxon rank‐sum test, one‐tailed, *p* = 0.202), genus (Wilcoxon rank‐sum test, one‐tailed, *p* = 0.088), or taxon (Wilcoxon rank‐sum test, one‐tailed, *p* = 0.266) level. There was, however, a significant difference at species level (Wilcoxon rank‐sum test, one‐tailed, *p* < 0.001; Figure 1b), with metabarcoding identifying 43 species, none of which could be identified through microscopy. Based on the overall taxa detected by the combined markers, metabarcoding detected 67.7% of the same families and 44.4% of the same genera as microscopy. When comparing the taxa identified in each sample, we found that metabarcoding detected an average of 53.2% (range 27%–77%) of the same plant families as microscopy in each honey and an average of 23.9% (range 9%–50%) of the same genera. We also compared only the top five families detected by microscopy with the combined top five families from each metabarcoding marker (resulting in six families for five of the samples where a family was detected by only one of the markers) and found that metabarcoding detected an average of three out of five of the most abundant families as microscopy, where most abundant refers to highest relative read abundance per sample for metabarcoding and highest pollen grain abundance per sample by microscopy (Table S3 and Figure S1). Microscopy was more consistent in detecting the main floral source nominated by producers, with all the nominated sources detected in the top five most abundant taxa of all 14 samples, compared to 12 with metabarcoding. Clustering analysis of microscopy data showed that trnL clustering produced very similar clusters, except a cluster including all SWA samples, was recovered only with the microscopy data (Figure S2).

## 4 DISCUSSION

Our results show that pollen DNA metabarcoding can produce detailed information regarding the floral sources of Australian honey, including inferences about its regional provenance. It can also be used to gain insights into the floral resources available to honeybees in the surrounding area. However, due to the lack of complete plant reference databases and poor taxonomic resolution of some taxa, the interpretation of metabarcoding results requires careful validation. With these considerations in mind, we suggest that pollen DNA metabarcoding has great potential as a molecular authentication method for Australian honey, and as a tool for hive monitoring programs to provide a thorough and continuous assessment of honeybee diet. Below we discuss some of the advantages and disadvantages of this approach.

### 4.1 Using pollen metabarcoding to examine honey composition

Previous pollen metabarcoding studies have shown that the combination of multiple markers provides more reliable than results than individual markers (Richardson, Lin, Quijia, et al., 2015). The use of two different metabarcoding markers allowed us to compare their relative performances when detecting plant taxa. Using both markers, we were able to detect between eight and 30 taxa per honey sample. However, the plastid trnL marker performed significantly better than ITS2 at detecting and identifying taxa. Its discriminating power tends to be less than ITS2 (Richardson et al., 2019), a longer marker with far more available sequences in public databases, and more reference sequences in our custom database (26.9% of recorded IBRA species in ITS2 database versus 15.2% in trnL database). A possible reason for this outcome is that the DNA in honey samples was highly degraded due to their long storage period, and amplification was more effective for the short target region of the trnL P6 loop. The trnL marker has been shown to be suitable for highly degraded DNA because of its short length and reliable amplification (Tab erlet et al., 2006). Other factors that may have influenced the difference in results between ITS2 and trnL are amplification biases, which can be taxon or marker‐specific (Deagle et al., 2014; Krehenwinkel et al., 2017; Moorhouse‐Gann et al., 2018). Potential biases toward some plant families have previously been detected in pollen metabarcoding (Hawkins et al., 2015). These factors again highlight the need to use multiple markers for more comprehensive detection of taxa.

We obtained valuable information from the honey samples to be able to differentiate them by major source region (East vs. West). The clustering analysis with trnL was also able to separate nearly all samples from the two major IBRA regions (Swan Coastal Plain and South East Queensland), demonstrating the potential of metabarcoding for providing geographic traceability and reflecting the composition of the surrounding floral community. Another major advantage of DNA metabarcoding is that as more reference sequences become available, it is possible to rerun an analysis using the same sequencing data to potentially identify more taxa. It is also relatively straightforward to amplify more markers from the same sample as long as sufficient DNA template is available. The laboratory protocol used here required only a few nanograms of DNA per marker, leaving enough extraction volume for further amplifications.

### 4.2 Pollen metabarcoding comparison with microscopy

Metabarcoding was able to detect many of the most abundant taxa identified with microscopy, often in similarly high proportions. Previous studies have found a positive correlation between the relative abundance of taxa identified in pollen samples by microscopy an metabarcoding, mostly for dominant taxa (Bänsch et al., 2020; Smart et al., 2017). The overlap in taxa identified by both methods was 21 families, representing 67.7% of the families detected by microscopy, and 12 genera, representing 44.4% of genera detected by microscopy. When comparing individual samples, the percentage overlap between microscopy and metabarcoding was 24% of genera on average. To an extent, these differences can be attributed to the different levels of resolution obtained from each method. However, some of the dissimilarities in taxa abundance between melissopalynology and metabarcoding have been recognized before and were considered partly due to the heterogeneous nature of honey and the sampling process (Hawkins et al., 2015).

Metabarcoding had similar issues as those described in the microscopy study with differentiating among taxa of some species‐rich families in Australia, such as Myrtaceae and Proteaceae. As with microscopy‐based analyses, a lack of segregating characters (i.e., sufficient nucleotide base differences) can limit the discriminating power of a metagenomics analysis. In this study, some common families had particularly poor discrimination, including Fabaceae, Asteraceae, and Myrtaceae. In particular, trnL had poor taxonomic resolution for Myrtaceae, with several *Eucalyptus, Angophora*, and *Corymbia* species sharing more than 99% sequence identity. The combination of both markers helped resolve some of these issues.

One of the advantages of DNA‐based methods in the study of bee diets is that traces of DNA could come from nectar plants, not just pollen, and these may be derived from different plant taxa. As shown by Prosser and Hebert (2017), the liquid constituent of honey contains a variety of plant sources that can be identified via metabarcoding. While the protocol for processing honey for DNA extraction requires filtering to concentrate pollen grains, it is likely that remnants of plant DNA from the liquid fraction remain in the pellet, and they can be detected by subsequent amplifications. However, quantification of pollen species content is not as reliable with metabarcoding as it is with melissopalynology. Therefore, for applications relying on accurate quantification of pollen content, a combination of a molecular profile and quantification via microscopy might be more suitable.

### 4.3 Foraging preferences of Australian honeybees

The most common taxa found in the diet of honeybees in the present study were Myrtaceae species, detected in all but one of the honeys. This was an expected result for nine of the honeys, as producers had specifically nominated one or more Myrtaceae taxa as the main foraging source, and it has been demonstrated that DNA metabarcoding can identify dominant pollen species in honey (Utzeri et al., 2018). The presence of *Eucalyptus* species in the majority of samples may also reflect their ubiquity and abundance in Australia. *Eucalyptus* species are commonly used foraging plants for honey production around the world (Bobis et al., 2020; Oddo et al., 2004). The *Eucalyptus* species diversity in Australian honey is, however, much higher than that of *Eucalyptus* honey produced elsewhere, as concluded by Sniderman et al. (2018). There are approximately 800 species of *Eucalyptus* in Australia, several of which are likely to be used by beekeepers. Additionally, the related genera *Corymbia* and *Angophora* are often collectively referred to as eucalypts by producers; therefore, correct identification of floral sources requires suitable examination methods. As with microscopy, metabarcoding also detected a high number of Myrtaceae types, but only two *Eucalyptus* could be identified to species level (*E. leucoxylon* and *E. microcorys*). *Eucalyptus* species have previously been shown to be difficult to differentiate even with the longer ITS2 marker (Prosser & Hebert, 2017). To determine which individual species are contributing to honeybee diet, better markers for *Eucalyptus* discrimination and identification are needed to improve detection.

Endemic Australian Proteaceae taxa such as *Grevillea* and *Macadamia* were significant components of some of the honeys examined. *Macadamia* was more prevalent in the eastern honeys (five eastern samples with relative read abundances >1%). *Macadamia* is cultivated commercially mostly in the eastern states of Australia, and its primary pollination vectors are honeybees and stingless native bees (Heard & Exley, 1994). *Grevillea* is a widespread and species‐rich genus in Australia, but is not commercially cultivated like *Macadamia*, thus more likely to be found in indigenous vegetation, while also commonly visited by honeybees (Taylor & Whelan, 1988). The eastern localities were much closer to large urban centers and are therefore more likely to contain non‐native species, while the west coast of Australia is less densely populated, with the southwest region in particular hosting a large number of endemic taxa (Hopper & Gioia, 2004). The results also confirmed that honeybees regularly forage on common weeds, as has been observed in other studies of intensely farmed areas (Requier et al., 2015), and may be contributing to the spread of some invasive species. *Trifolium* species are more common in the eastern states and were prominent components of the South East Queensland honeys and one honey from Brigalow Belt South (SJ9). One of the species detected by ITS2, *T. repens,* or white clover, is commonly cultivated in Australia for pasture and is pollinated by honeybees (Goodman & Williams, 1994; Lane et al., 2000). While it is also found in Western Australia, its presence in eastern honey samples may indicate the proximity of hives to grazing lands.

One of the first challenges toward obtaining a more accurate identification of the foraging preferences of honeybees, in particular Australian honeybees, will be to increase the number of available reference sequences for endemic taxa. While many introduced species are present in global sequence databases, much of the Australian native flora remains poorly characterized, apart from a few taxa (Dormontt et al., 2018). Availability of comprehensive and well‐curated reference sequences can highly improve the performance of metabarcoding analyses. Our recommendation, as previously proposed by others (Dormontt et al., 2018), is that this need should be addressed with comprehensive efforts to generate more sequences, preferably from multiple plant markers, and making use of available resources such as herbarium specimens.

## 5 CONCLUSIONS

In this study, we have shown that pollen metabarcoding is a powerful tool that can be used to characterize honey content, can potentially determine its provenance, and can inform us on the foraging preferences of honeybees. Metabarcoding was able to identify significantly more species than microscopy and could be used as a stand‐alone tool to provide a botanical profile of honey. Furthermore, the use of metabarcoding in monitoring the diets of honeybees could help determine whether they have access to adequate floral resources, which is one of the recommendations for the management of hive health.

## CONFLICT OF INTEREST

The authors declare no conflict of interest.

## AUTHOR CONTRIBUTIONS

**Liz Milla:** Conceptualization (equal); Data curation (lead); Formal analysis (equal); Software (lead); Writing‐original draft (lead); Writing‐review & editing (equal). **Kale Sniderman:** Conceptualization (supporting); Formal analysis (supporting); Resources (equal); Writing‐review & editing (equal). **Rose Lines:** Data curation (equal); Resources (supporting); Writing‐review & editing (supporting). **Mahsa Mousavi‐Derazmahalleh:** Data curation (supporting); Software (supporting); Writing‐review & editing (supporting). **Francisco Encinas‐Viso:** Conceptualization (equal); Formal analysis (equal); Writing‐review & editing (equal).

## Supporting information

Supplementary Material

Figure S1

Figure S2

## Data Availability

Raw sequences and the final dataset are available on the CSIRO Data Access Portal (https://data.csiro.au/collections/collection/CIcsiro:50306).
