# Supplementary material for: Pollen DNA metabarcoding identifies regional provenance and high plant diversity in Australian honey
Source: Ecol Evol. 2021 Jun 3;11(13):8683–98. doi: 10.1002/ece3.7679 (PMC8258210; doi:10.1002/ece3.7679)
Supplement: Supplementary file 1 — Supplementary Material [file ECE3-11-8683-s001.docx]

**Pollen DNA metabarcoding identifies regional provenance and high plant diversity in Australian honey - supplementary tables and figures**

**Table S1.** Mapping of original taxon name detected by microscopy from Sniderman et al (2018) to family, genus, species and taxon names used in this study.

| **Original taxon name (Sniderman et al 2018)** | **Family** | **Genus** | **Species** | **Taxon** |
| --- | --- | --- | --- | --- |
| Eucalyptus_macrorhyncha | Myrtaceae | Eucalyptus | macrorhyncha | Eucalyptus_macrorhyncha |
| Eucalyptus | Myrtaceae | Eucalyptus |  | Eucalyptus_sp |
| Echium | Boraginaceae | Echium |  | Echium_sp |
| Brassicaceae | Brassicaceae |  |  | Brassicaceae_sp |
| Corymbia cf | Myrtaceae | Corymbia |  | Corymbia_sp |
| Leptospermeae cf | Myrtaceae |  |  | Leptospermeae_sp |
| Myrtaceae undif. | Myrtaceae |  |  | Myrtaceae_sp |
| Fabaceae undif. | Fabaceae |  |  | Fabaceae_sp |
| Macadamia | Proteaceae | Macadamia |  | Macadamia_sp |
| Asteraceae tubuliflorae | Asteraceae |  |  | Tubuliflorae_sp |
| Acacia | Fabaceae | Acacia |  | Acacia_sp |
| Vicia cf | Fabaceae | Vicia |  | Vicia_sp |
| Trifolium, reticulate | Asteraceae | Trifolium |  | Trifolium_sp |
| Banksia | Proteaceae | Banksia |  | Banksia_sp |
| Eucryphia | Cunoniaceae | Eucryphia |  | Eucryphia_sp |
| Myoporeae, large | Myoporaceae |  |  | Myoporeae_spLarge |
| Monosulcate monocot | Undetermined |  |  | Undetermined_spMonocot |
| Myoporeae, small | Myoporaceae |  |  | Myoporeae_spSmall |
| Bursaria | Pittosporaceae | Bursaria |  | Bursaria_sp |
| Elaeocarpaceae | Elaeocarpaceae |  |  | Elaeocarpaceae_sp |
| Asteraceae Cichoroideae | Asteraceae |  |  | Cichoroideae_sp |
| Leucopogon | Ericaceae | Leucopogon |  | Leucopogon_sp |
| Rosaceae cf | Rosaceae |  |  | Rosaceae_sp |
| Rutaceae cf | Rutaceae |  |  | Rutaceae_sp |
| Oleaceae | Oleaceae |  |  | Oleaceae_sp |
| Trifolium repens cf, psilate | Asteraceae | Trifolium | repens | Trifolium_repens |
| monosulcate reticulate | Undetermined |  |  | Undetermined_spReticulate |
| Centaurea type | Asteraceae | Centaurea |  | Centaurea_spCentaurea |
| Prunus cf | Rosaceae | Prunus |  | Prunus_sp |
| Bossiaeae/Hibbertia | Dilleniaceae | Hibbertia |  | Hibbertia_sp |
| Dodonaea | Sapindaceae | Dodonaea |  | Dodonaea_sp |
| Sapindaceae reticulate | Sapindaceae |  |  | Sapindaceae_spReticulate |
| Citrus | Rutaceae | Citrus |  | Citrus_sp |
| Lotus cf | Nelumbonaceae | Lotus |  | Lotus_sp |
| Caryophyllaceae | Caryophyllaceae |  |  | Caryophyllaceae_sp |
| Rhamnaceae tiny | Rhamnaceae |  |  | Rhamnaceae_spTiny |
| Ranunculus cf | Ranunculaceae | Ranunculus |  | Ranunculus_sp |
| Celtis | Cannabaceae | Celtis |  | Celtis_sp |
| Proteaceae undif. | Proteaceae |  |  | Proteaceae_sp |
| Sarcozona cf | Aizoaceae | Sarcozona |  | Sarcozona_sp |
| Anacardiaceae | Anacardiaceae |  |  | Anacardiaceae _sp |
| Lamiaceae | Lamiaceae |  |  | Lamiaceae_sp |
| Medicago | Asteraceae | Medicago |  | Medicago_sp |
| Carpobrotus | Aizoaceae | Carpobrotus |  | Carpobrotus_sp |
| Asteraceae, large oblate | Asteraceae |  |  | Asteraceae_spLargeOblate |
| Goodeniaceae | Goodeniaceae |  |  | Goodeniaceae_sp |
| Pomaderris type | Rhamnaceae | Pomaderris |  | Pomaderris_sp |
| Rhamnaceae big | Rhamnaceae |  |  | Rhamnaceae_spBig |
| Apiaceae | Apiaceae |  |  | Apiaceae_sp |
| Cassia cf | Lauraceae | Cassia |  | Cassia_sp |
| Hakea/Grevillea | Proteaceae | Grevillea |  | Grevillea_sp |
| Polygonum cf | Polygonaceae | Polygonum |  | Polygonum_sp |
| Rubus cf | Rosaceae | Rubus |  | Rubus_sp |
| Pimelea | Thymelaeaceae | Pimelea |  | Pimelea_sp |
| Ligustrum cf | Oleaceae | Ligustrum |  | Ligustrum_sp |
| Vitis | Vitaceae | Vitis |  | Vitis_sp |
| Loranthaceae | Loranthaceae |  |  | Loranthaceae_sp |
| Euphorbiaceae | Euphorbiaceae |  |  | Euphorbiaceae _sp |
| Lily-like monocot | Liliaceae |  |  | Liliaceae_sp |
| Salix | Salicaceae | Salix |  | Salix_sp |
| Anacardiaceae -Mangifera cf | Anacardiaceae | Mangifera |  | Mangifera_sp |
| Daucus cf, protruding pores | Apiaceae | Daucus |  | Daucus_sp |
| Monotoca | Ericaceae | Monotoca |  | Monotoca_sp |
| Scroph/Lamiaceae type (large spheroid reticulate) | Lamiaceae |  |  | Lamiaceae_spScroph |
| Cunoniaceae (3cp) | Cunoniaceae |  |  | Cunoniaceae_sp3cp |
| Ericaceae tetrad | Ericaceae |  |  | Ericaceae_spTetrad |

**Table S2**. Taxa detected in all honey metabarcoding samples with ITS2 and trnL. Total reads are for all samples combined for each marker.

| **Family** | **Genus** | **Species** | **Taxon** | **Total  ITS2 reads** | **Total  trnL reads** |
| --- | --- | --- | --- | --- | --- |
| Amaryllidaceae | Allium | Allium cepa | Allium cepa | 60 | 0 |
| Asteraceae | Arctotheca |  | Arctotheca sp | 8512 | 0 |
| Asteraceae |  |  | Asteraceae sp | 161546 | 40696 |
| Boraginaceae | Echium | Echium vulgare | Echium vulgare | 2031 | 0 |
| Boraginaceae |  |  | Boraginaceae sp | 0 | 1361 |
| Brassicaceae | Brassica | Brassica napus | Brassica napus | 1594 | 0 |
| Brassicaceae | Brassica |  | Brassica sp | 128656 | 0 |
| Brassicaceae |  |  | Brassicaceae sp | 130265 | 116077 |
| Brassicaceae | Rapistrum |  | Rapistrum sp | 55 | 0 |
| Brassicaceae | Diplotaxis | Diplotaxis tenuifolia | Diplotaxis tenuifolia | 0 | 11103 |
| Cucurbitaceae |  |  | Cucurbitaceae sp | 906 | 0 |
| Fabaceae | Daviesia | Daviesia divaricata | Daviesia divaricata | 6112 | 0 |
| Fabaceae |  |  | Fabaceae sp | 11162 | 161312 |
| Fabaceae | Glycine | Glycine max | Glycine max | 21715 | 0 |
| Fabaceae | Glycine |  | Glycine sp | 209 | 0 |
| Fabaceae | Jacksonia | Jacksonia horrida | Jacksonia horrida | 2094 | 0 |
| Fabaceae | Pisum |  | Pisum sp | 1878 | 0 |
| Fabaceae | Trifolium | Trifolium hirtum | Trifolium hirtum | 190 | 0 |
| Fabaceae | Trifolium | Trifolium repens | Trifolium repens | 8704 | 0 |
| Fabaceae | Trifolium |  | Trifolium sp | 3378 | 98218 |
| Fabaceae | Aotus | Aotus subglauca | Aotus subglauca | 0 | 403 |
| Fabaceae | Austrocallerya | Austrocallerya australis | Austrocallerya australis | 0 | 12472 |
| Fabaceae | Cytisus | Cytisus scoparius | Cytisus scoparius | 0 | 321 |
| Fabaceae | Daviesia | Daviesia ulicifolia | Daviesia ulicifolia | 0 | 2220 |
| Fabaceae | Gleditsia | Gleditsia triacanthos | Gleditsia triacanthos | 0 | 1718 |
| Fabaceae | Hardenbergia |  | Hardenbergia sp | 0 | 2413 |
| Fabaceae | Leucaena | Leucaena leucocephala | Leucaena leucocephala | 0 | 1956 |
| Fabaceae | Medicago | Medicago sativa | Medicago sativa | 0 | 2834 |
| Fabaceae | Neonotonia | Neonotonia wightii | Neonotonia wightii | 0 | 2155 |
| Fabaceae | Robinia | Robinia pseudoacacia | Robinia pseudoacacia | 0 | 9 |
| Fabaceae | Vicia |  | Vicia sp | 0 | 32116 |
| Myrtaceae | Corymbia |  | Corymbia sp | 76040 | 0 |
| Myrtaceae | Eucalyptus | Eucalyptus leucoxylon | Eucalyptus leucoxylon | 57 | 0 |
| Myrtaceae | Eucalyptus | Eucalyptus microcorys | Eucalyptus microcorys | 588 | 0 |
| Myrtaceae | Eucalyptus |  | Eucalyptus sp | 575081 | 0 |
| Myrtaceae | Kunzea | Kunzea ericoides | Kunzea ericoides | 557 | 0 |
| Myrtaceae |  |  | Myrtaceae sp | 201686 | 277820 |
| Myrtaceae | Melaleuca | Melaleuca nodosa | Melaleuca nodosa | 0 | 41588 |
| Plantaginaceae | Plantago | Plantago lanceolata | Plantago lanceolata | 69888 | 8664 |
| Proteaceae | Macadamia |  | Macadamia sp | 7836 | 0 |
| Proteaceae | Grevillea |  | Grevillea sp | 0 | 21985 |
| Proteaceae | Macadamia | Macadamia integrifolia | Macadamia integrifolia | 0 | 24511 |
| Proteaceae |  |  | Proteaceae sp | 0 | 45452 |
| Salicaceae | Populus | Populus deltoides | Populus deltoides | 60022 | 0 |
| Salicaceae |  |  | Salicaceae sp | 0 | 3247 |
| Adoxaceae | Viburnum |  | Viburnum sp | 0 | 9492 |
| Aizoaceae | Lampranthus | Lampranthus spectabilis | Lampranthus spectabilis | 0 | 4431 |
| Anacardiaceae | Mangifera | Mangifera indica | Mangifera indica | 0 | 1105 |
| Anacardiaceae | Schinus | Schinus molle | Schinus molle | 0 | 1073 |
| Asphodelaceae | Eremurus | Eremurus spectabilis | Eremurus spectabilis | 0 | 213 |
| Bignoniaceae |  |  | Bignoniaceae sp | 0 | 1357 |
| Cannabaceae |  |  | Cannabaceae sp | 0 | 18057 |
| Cannabaceae | Cannabis | Cannabis sativa | Cannabis sativa | 0 | 12 |
| Casuarinaceae | Casuarina |  | Casuarina sp | 0 | 17459 |
| Celastraceae |  |  | Celastraceae sp | 0 | 63 |
| Chenopodiaceae | Chenopodium |  | Chenopodium sp | 0 | 162 |
| Commelinaceae | Callisia |  | Callisia sp | 0 | 387 |
| Convolvulaceae |  |  | Convolvulaceae sp | 0 | 2269 |
| Cordiaceae |  |  | Cordiaceae sp | 0 | 149 |
| Cupressaceae | Callitris | Callitris glaucophylla | Callitris glaucophylla | 0 | 28 |
| Cyperaceae | Mesomelaena | Mesomelaena pseudostygia | Mesomelaena pseudostygia | 0 | 15 |
| Elaeocarpaceae | Elaeocarpus |  | Elaeocarpus sp | 0 | 5965 |
| Ericaceae | Brachyloma | Brachyloma daphnoides | Brachyloma daphnoides | 0 | 50 |
| Ericaceae | Brachyloma |  | Brachyloma sp | 0 | 156 |
| Ericaceae |  |  | Ericaceae sp | 0 | 218 |
| Euphorbiaceae | Adriana | Adriana tomentosa | Adriana tomentosa | 0 | 24 |
| Euphorbiaceae | Euphorbia |  | Euphorbia sp | 0 | 930 |
| Euphorbiaceae |  |  | Euphorbiaceae sp | 0 | 563 |
| Euphorbiaceae | Mallotus |  | Mallotus sp | 0 | 20602 |
| Geraniaceae | Pelargonium |  | Pelargonium sp | 0 | 6529 |
| Goodeniaceae | Scaevola |  | Scaevola sp | 0 | 1060 |
| Gyrostemonaceae |  |  | Gyrostemonaceae sp | 0 | 91 |
| Haemodoraceae | Macropidia | Macropidia fuliginosa | Macropidia fuliginosa | 0 | 17 |
| Heliotropiaceae | Heliotropium |  | Heliotropium sp | 0 | 1572 |
| Iridaceae | Crocus |  | Crocus sp | 0 | 1005 |
| Juglandaceae | Carya |  | Carya sp | 0 | 3703 |
| Lauraceae |  |  | Lauraceae sp | 0 | 10649 |
| Malvaceae | Abutilon | Abutilon theophrasti | Abutilon theophrasti | 0 | 69 |
| Oleaceae |  |  | Oleaceae sp | 0 | 5213 |
| Oxalidaceae | Oxalis |  | Oxalis sp | 0 | 435 |
| Papaveraceae | Argemone | Argemone mexicana | Argemone mexicana | 0 | 8 |
| Papaveraceae | Papaver | Papaver somniferum | Papaver somniferum | 0 | 172 |
| Passifloraceae | Passiflora |  | Passiflora sp | 0 | 7638 |
| Pinaceae |  |  | Pinaceae sp | 0 | 519 |
| Poaceae | Ehrharta |  | Ehrharta sp | 0 | 77 |
| Poaceae |  |  | Poaceae sp | 0 | 1051 |
| Ranunculaceae | Clematis |  | Clematis sp | 0 | 20 |
| Ranunculaceae | Ranunculus |  | Ranunculus sp | 0 | 2323 |
| Restionaceae | Lyginia | Lyginia barbata | Lyginia barbata | 0 | 175 |
| Rhamnaceae | Alphitonia |  | Alphitonia sp | 0 | 2681 |
| Rosaceae | Prunus | Prunus mahaleb | Prunus mahaleb | 0 | 127 |
| Rosaceae |  |  | Rosaceae sp | 0 | 3720 |
| Rutaceae |  |  | Rutaceae sp | 0 | 299 |
| Sapindaceae | Dodonaea | Dodonaea viscosa | Dodonaea viscosa | 0 | 3513 |
| Sapindaceae |  |  | Sapindaceae sp | 0 | 1687 |
| Scrophulariaceae |  |  | Scrophulariaceae sp | 0 | 4636 |
| Solanaceae | Capsicum |  | Capsicum sp | 0 | 3387 |
| Solanaceae |  |  | Solanaceae sp | 0 | 9825 |
| Theaceae | Pyrenaria | Pyrenaria spectabilis | Pyrenaria spectabilis | 0 | 330 |
| Thymelaeaceae | Pimelea |  | Pimelea sp | 0 | 830 |
| Ulmaceae | Ulmus | Ulmus procera | Ulmus procera | 0 | 2810 |
| Verbenaceae |  |  | Verbenaceae sp | 0 | 8556 |
| Vitaceae |  |  | Vitaceae sp | 0 | 60 |

**Table S3**. Top five most abundant families detected in each honey sample by metabarcoding or microscopy (from Sniderman et al 2018). Most abundant family refers to the highest proportion of reads (metabarcoding) or pollen grains (microscopy) combined for all taxa per family in each sample.

| **Region** | **ID** | **Producer’s description** | **Top 5 families detected by metabarcoding** | **Top 5 families detected by microscopy** | **Overlap between methods** |
| --- | --- | --- | --- | --- | --- |
| West | SJ01 | Spring Eucalypt, WA | Brassicaceae, Fabaceae, Geraniaceae, Myrtaceae, Proteaceae | Ericaceae, Fabaceae, Myrtaceae, Proteaceae | Fabaceae, Myrtaceae, Proteaceae |
| West | SJ02 | Mild spring Eucalypt, WA | Brassicaceae, Casuarinaceae, Fabaceae, Myrtaceae, Proteaceae | Ericaceae, Fabaceae, Myrtaceae, Proteaceae | Fabaceae, Myrtaceae, Proteaceae |
| West | SJ03 | Spring Eucalypt, WA | Asteraceae, Brassicaceae, Fabaceae, Myrtaceae, Proteaceae | Boraginaceae, Myrtaceae, Proteaceae | Myrtaceae, Proteaceae |
| West | SJ04 | Spring Eucalypt, WA | Brassicaceae, Fabaceae, Myrtaceae, Proteaceae | Brassicaceae, Myrtaceae, Oleaceae, Proteaceae | Brassicaceae, Myrtaceae, Proteaceae |
| West | SJ05 | Spring Eucalypt, WA | Asteraceae, Brassicaceae, Fabaceae, Myrtaceae, Plantaginaceae, Proteaceae | Brassicaceae, Fabaceae, Myrtaceae, Proteaceae | Brassicaceae, Fabaceae, Myrtaceae, Proteaceae |
| East | SJ06 | Yapunyah | Asteraceae, Casuarinaceae, Fabaceae, Myrtaceae, Scrophulariaceae | Asteraceae, Brassicaceae, Myoporaceae, Myrtaceae | Asteraceae, Myrtaceae |
| East | SJ07 | Canola | Brassicaceae, Fabaceae, Myrtaceae | Asteraceae, Brassicaceae, Fabaceae, Myrtaceae | Brassicaceae, Fabaceae, Myrtaceae |
| East | SJ08 | Yapunyah | Asteraceae, Brassicaceae, Fabaceae, Myrtaceae, Scrophulariaceae | Asteraceae, Brassicaceae, Myoporaceae, Myrtaceae | Asteraceae, Brassicaceae, Myrtaceae |
| East | SJ09 | Mixed Honey | Amaryllidaceae, Brassicaceae, Fabaceae, Myrtaceae, Solanaceae | Brassicaceae, Fabaceae, Goodeniaceae, Myrtaceae, Polygonaceae | Brassicaceae, Fabaceae, Myrtaceae |
| East | SJ10 | Macadamia | Brassicaceae, Euphorbiaceae, Fabaceae, Proteaceae, Verbenaceae | Asteraceae, Brassicaceae, Fabaceae, Myrtaceae, Proteaceae | Brassicaceae, Fabaceae, Proteaceae |
| East | SJ11 | White Box/Mixed honey | Asteraceae, Fabaceae, Myrtaceae, Oleaceae, Solanaceae | Asteraceae, Brassicaceae, Myrtaceae | Asteraceae, Myrtaceae |
| East | SJ12 | Blue Gum/Mixed Honey | Brassicaceae, Cannabaceae, Myrtaceae, Verbenaceae | Fabaceae, Myrtaceae, Proteaceae, Rutaceae, Undetermined | Myrtaceae |
| East | SJ13 | Macadamia | Asteraceae, Lauraceae, Passifloraceae, Proteaceae, Solanaceae | Asteraceae, Fabaceae, Myrtaceae, Proteaceae | Asteraceae, Proteaceae |
| East | SJ14 | Mixed Honey/Macadamia | Adoxaceae, Asteraceae, Fabaceae, Myrtaceae | Asteraceae, Elaeocarpaceae, Fabaceae, Myrtaceae, Proteaceae | Asteraceae, Fabaceae, Myrtaceae |
| East | SJ15 | Macadamia | Asteraceae, Euphorbiaceae, Fabaceae, Plantaginaceae, Salicaceae | Asteraceae, Fabaceae, Myrtaceae, Proteaceae | Asteraceae, Fabaceae |


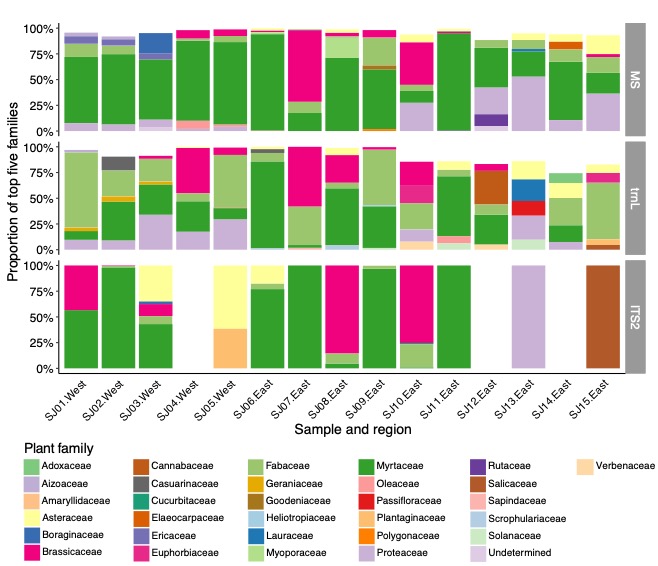


**Figure S1**. Proportion of top five families by relative abundance of either total pollen grains (microscopy) or total reads (metabarcoding). Top panel shows microscopy (based on Sniderman et al, 2018), middle panel shows trnL and bottom panel shows ITS2 results. Colours are used to differentiate families.

**Figure S2**. Heatmap of beta diversity and clustering analysis for microscopy data. Cells are coloured by relative read abundance, and row dendrogram tips are colour coded by IBRA regions. Plant taxa (X axis) are filtered to those present in abundances > 0.1%.
