# Supplementary figures and images for: Pollen DNA metabarcoding identifies regional provenance and high plant diversity in Australian honey
Source: Ecol Evol. 2021 Jun 3;11(13):8683–98. doi: 10.1002/ece3.7679 (PMC8258210; doi:10.1002/ece3.7679)

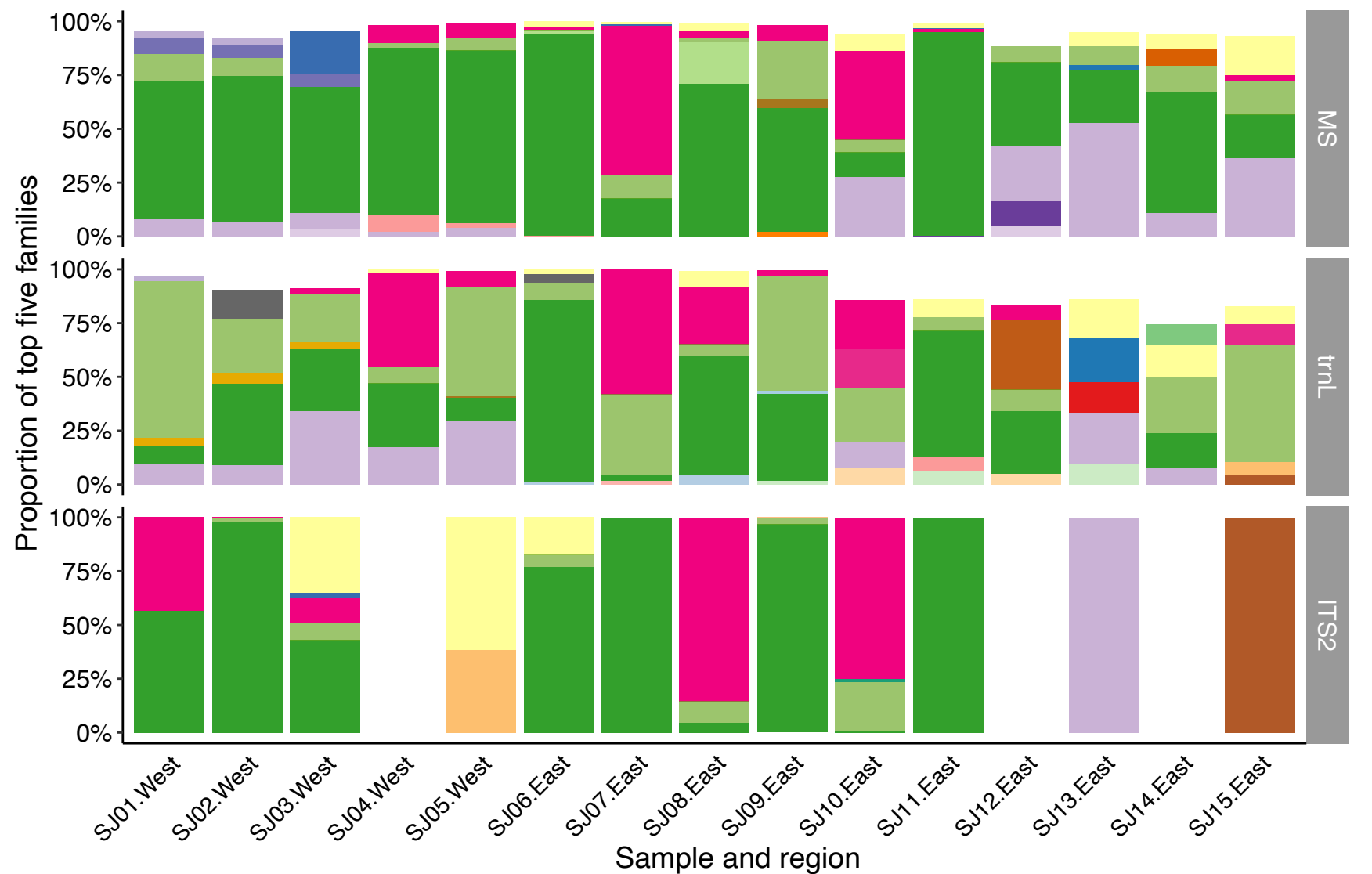

### Plant family

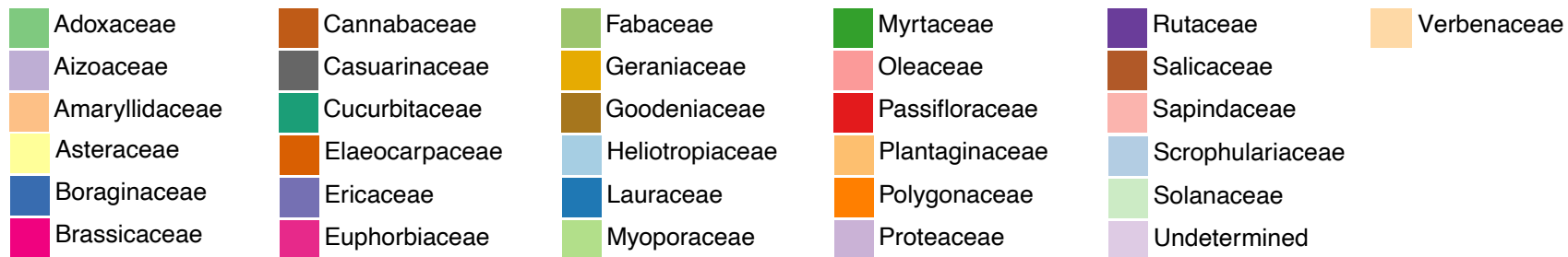

Supplement: Supplementary file 2 — Figure S1 [file ECE3-11-8683-s002.pdf]

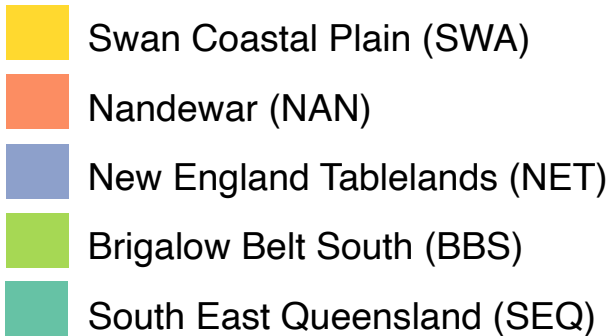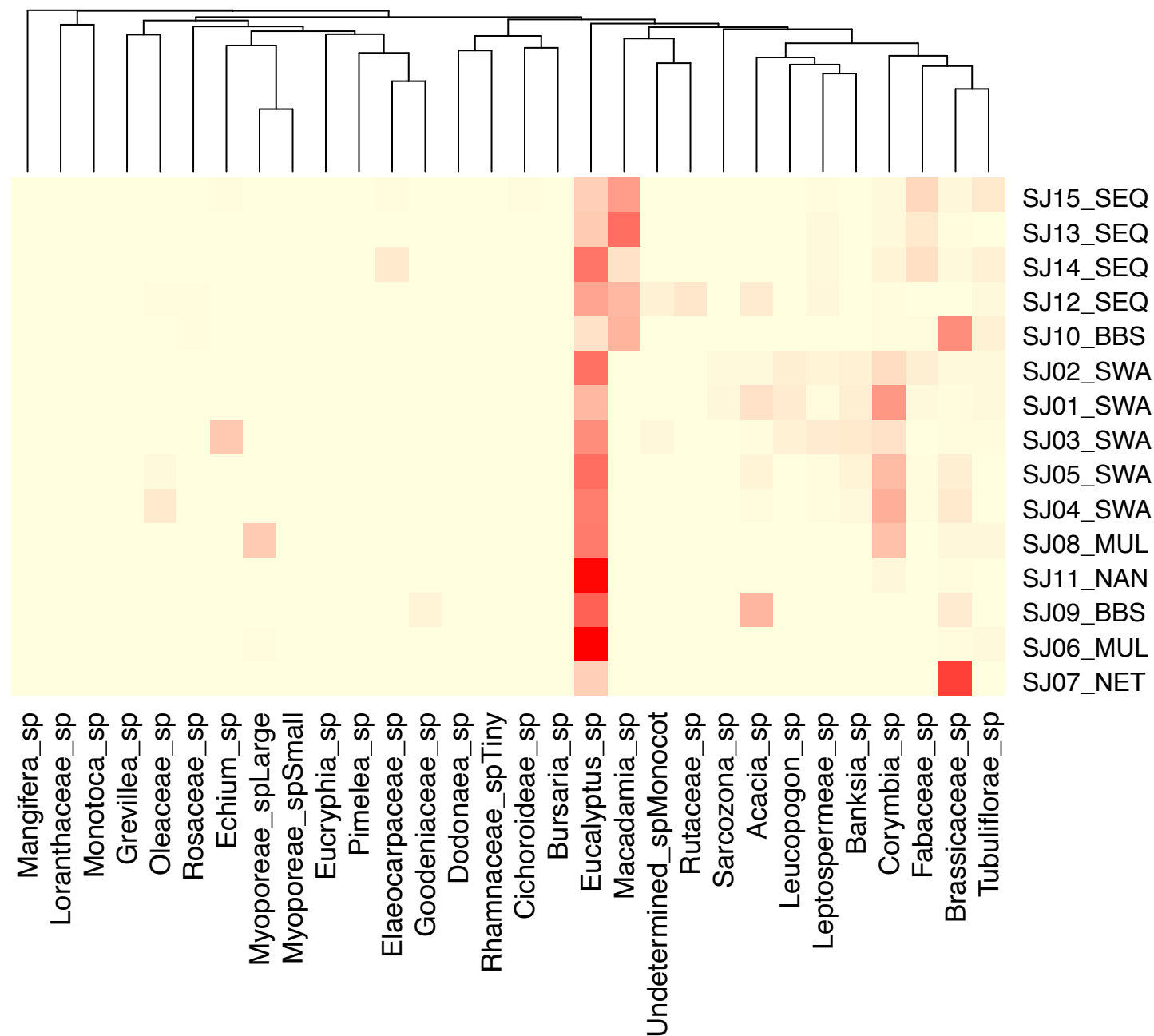

Supplement: Supplementary file 3 — Figure S2 [file ECE3-11-8683-s003.pdf]
